# P-1179. Evaluation of a Novel Citrus Silver Nasal Cleansing Swab on Staphylococcus aureus Microbial Activity in Porcine Nares

**DOI:** 10.1093/ofid/ofaf695.1372

**Published:** 2026-01-11

**Authors:** Caitlin Crews-Stowe

**Affiliations:** Scenic City Healthcare Consulting, Chattanooga, Tennessee

## Abstract

**Background:**

*Staphylococcus aureus* is one of the most common pathogens for many healthcare associated infections including surgical site infections and central-line associated bloodstream infections. Nasal decolonization has been previously found to be an effective strategy for reducing HAIs in conjunction with a bathing strategy, but there are limited decolonization options for providers to choose from. An en-vivo study utilizing pig nares was performed to evaluate the efficacy of a novel, citrus silver nasal cleansing swab on Methicillin-Sensitive *Staphylococcus aureus (MSSA).*

**Figure:**
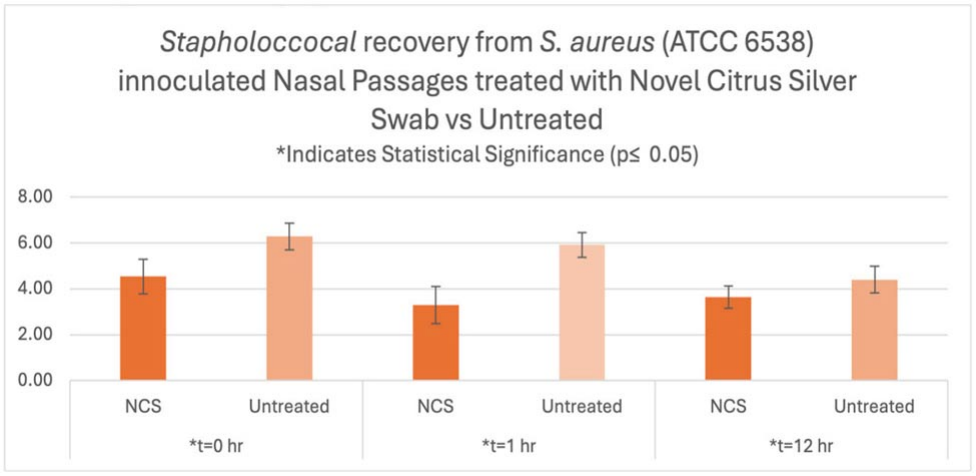
Recovery from Staphylococcus aureus inoculated nares

**Methods:**

A prospective cohort study consisting of two groups, an untreated control and a treatment group (n=3 per group), was performed in pigs. 10^8^ to 10^9^ logs of *Staphylococcus aureus* was inoculated into the pigs' nares and allowed to incubate for 15 minutes. After the incubation period, the treatment group received a 15 second application into each nare with the novel citrus silver nasal swab. The pigs nares in both groups were then sampled and tested at timepoints 0 hour, 1 hour, and 12 hours. The samples were incubated for 72 hours and quantitative results were reported. Statistical analyses were performed to determine the percent reduction and differences in microbial burden individually and between the groups. All research was performed according to Good Clinical Laboratory Practices and the International Standards for the Care and Use of Laboratory Animals.

**Results:**

A statistically significant 97.74% reduction (p= 0.0012) of MSSA was seen at the 0 minute mark in the treatment group vs. the untreated control group. There were also statistically significant reductions of 99.8% and 99.78% at the 1 hour(p< 0.0001) and 12 hour (p=0.033) mark in the treatment group compared to the control group, respectively.

**Conclusion:**

The study found that the citrus silver nasal cleansing swab significantly reduced MSSA within a few minutes of application and showed a significant sustained reduction of MSSA for 12 hours. Further research in human subjects is underway.

**Disclosures:**

Caitlin Crews-Stowe, MPH, CPH, CIC, CPHQ, VA-BC, Avadim Health: Advisor/Consultant|MPM Medical: Advisor/Consultant

